# The public health impact of economic fluctuations in a Latin American country: mortality and the business cycle in Colombia in the period 1980–2010

**DOI:** 10.1186/s12939-015-0176-9

**Published:** 2015-05-27

**Authors:** Ivan Arroyave, Philipp Hessel, Alex Burdorf, Jesus Rodriguez-Garcia, Doris Cardona, Mauricio Avendaño

**Affiliations:** Department of Public Health, Erasmus University Medical Center, Dr. Molewaterplein 50, GE 3015 Rotterdam, The Netherlands; National School of Public Health, University of Antioquia, Calle 62 N° 52-59, Medellín, Colombia; London School of Economics and Political Science, LSE Health and Social Care, Cowdray House Houghton Street, London, WC2A 2AE UK; Department of Social and Behavioral Sciences, Harvard School of Public Health, 677 Huntington Avenue, Boston, MA 02115 USA; Pontificia Universidad Javeriana, Carrera 7 # 40-62, DC 11001000 Bogotá, Colombia; Faculty of Medicine, Universidad CES, Calle 10A # 22-04, Medellín, Colombia

**Keywords:** Mortality, Economic recession, Colombia, Developing countries, Health insurance

## Abstract

**Introduction:**

Studies in high-income countries suggest that mortality is related to economic cycles, but few studies have examined how fluctuations in the economy influence mortality in low- and middle-income countries. We exploit regional variations in gross domestic product per capita (GDPpc) over the period 1980–2010 in Colombia to examine how changes in economic output relate to adult mortality.

**Methods:**

Data on the number of annual deaths at ages 20 years and older (*n* = 3,506,600) from mortality registries, disaggregated by age groups, sex and region, were linked to population counts for the period 1980–2010. We used region fixed effect models to examine whether changes in regional GDPpc were associated with changes in mortality. We carried out separate analyses for the periods 1980–1995 and 2000–2010 as well as by sex, distinguishing three age groups: 20–44 (predominantly young working adults), 45–64 (middle aged working adults), and 65+ (senior, predominantly retired individuals).

**Results:**

The association between regional economic conditions and mortality varied by period and age groups. From 1980 to 1995, increases in GDPpc were unrelated to mortality at ages 20 to 64, but they were associated with reductions in mortality for senior men. In contrast, from 2000 to 2010, changes in GDPpc were not associated with old age mortality, while an increase in GDPpc was associated with a decline in mortality at ages 20–44 years. Analyses restricted to regions with high registration coverage yielded similar albeit less precise estimates for most sub-groups.

**Conclusions:**

The relationship between business cycles and mortality varied by period and age in Colombia. Most notably, mortality shifted from being acyclical to being countercyclical for males aged 20–44, while it shifted from being countercyclical to being acyclical for males aged 65+.

## Resumen

**Introducción:**

Estudios previos en países de altos ingresos indican que la mortalidad está relacionada con los ciclos económicos, pero pocos estudios han examinado cómo las fluctuaciones en la economía afectan la mortalidad en países de ingresos medios y bajos. Este estudio explota las variaciones regionales del Producto Interno Bruto per cápita (PIBpc) en el período 1980–2010 en Colombia para examinar cómo los cambios en el ciclo económico se relacionan con la mortalidad en adultos.

**Métodos:**

Los datos sobre el número anual de muertes en población mayor de 20 años (*n* = 3.506.600), fueron agregados por grupos quinquenales de edad, sexo y región a partir de los registros individuales de mortalidad y se cruzaron con conteos de población para el período 1980–2010. Empleamos modelos de efectos fijos regionales para examinar si los cambios en el PIBpc regional se asocian con las oscilaciones en la mortalidad. Se analizaron por separado los períodos 1980–1995 y 2000–2010, así como por sexo, distinguiendo tres grupos de edad: 20 a 44 (que representan predominantemente jóvenes trabajadores), 45 a 64 (adultos trabajadores de mediana edad), y 65 años o mayores (adultos mayores en edad de jubilación).

**Resultados:**

La asociación entre las condiciones económicas regionales y la mortalidad varían según el periodo analizado y la edad. De 1980 a 1995, los cambios en el PIBpc no se relacionaron con la mortalidad en edades de 20 a 64, pero se asociaron con reducciones de la mortalidad para los hombres mayores. Por el contrario, de 2000 a 2010, los cambios en PIBpc no se asociaron con la mortalidad en personas mayores, mientras que un aumento en PIBpc se asoció con una disminución de la mortalidad en las edades 20 a 44 años. La cobertura del registro de mortalidad mejoró con el tiempo; los análisis restringidos a regiones con mayor registro produjeron estimaciones similares, aunque con grandes errores estándar.

**Conclusiones:**

La relación entre los ciclos económicos y la mortalidad en Colombia varía según el período y los grupos de edad estudiados. Más importante aún, la mortalidad en hombres pasó de ser acíclica a contracíclica en el grupo de 20–44 años de edad, mientras que pasó de ser contracíclica a acíclica para los mayores de 65 años de edad.

**Palabras clave:**

Mortalidad; Recesión económica; Colombia; Países en desarrollo; Seguro de Salud.

## Introduction

A series of studies in high-income countries found that mortality is procyclical — it decreases when the economy contracts and increases when the economy expands [1–12]. A common interpretation of this finding is that during times of intense economic activity, individuals have less flexibility in making time allocation decisions, leading to behavioral changes such as declines in the time spent exercising and cooking healthy foods, or scheduling medical appointments [1, 13]. Recent evidence suggests, however, that this relationship may not hold for recent periods in high-income countries [12]. In addition, little is known about this relationship in low- or middle-income countries, with the few studies conducted yielding contradictory findings [14–17].

A difference in the relationship between economic cycles and mortality between high- and low- or middle-income countries may exist for several reasons. High-income countries have well-developed social safety nets and healthcare systems [18, 19]. Given that most low- and middle-income countries still lack comparable systems, their populations may be more vulnerable to the negative effects of economic downturns [20]. Furthermore, in high-income countries, non-communicable diseases are believed to be important drivers of the association between business cycles and mortality [21]. While non-communicable diseases are increasingly important in low- and middle-income countries, communicable diseases and injuries remain an important cause of death in Colombia [22]. Many of these causes of death are amenable to medical intervention, suggesting that not only non-communicable disease risk factors but also health protection systems may be important drivers of a potential relationship between mortality and the economy. As a result, the relationship may be different for low- and middle-income countries.

The purpose of this study is to assess the association between regional economic conditions and mortality in the periods 1980–1995 and 2000–2010. We follow the approach of previous studies and exploit regional variations over time in economic conditions [1, 5, 21] to provide further insights into this relationship in a middle-income, Latin American country. Colombia offers a unique setting to assess this relationship due to several reasons. Gross domestic product (GDP) almost tripled from 1980 to 2010, yet Colombia witnessed major oscillations in the economy with troughs in real GDP growth in 1982, 1991 and 1999, and peaks in 1986, 1995 and 2007 [23]. Since the early 1990’s, Colombia also initiated a major health care reform culminating in the introduction of mandatory health insurance coverage in 1993 [24]. The reform assigned citizens to either a contributory scheme (for employed workers and their families) or a subsidized scheme (for poor individuals not in formal employment and their families) and led to an increase in health insurance coverage from 23.7 % in 1993, to 93.4 % by 2009 [25], mostly attributable to an increase in subsidized insurance coverage from 2000 onwards [24]. In addition, social expenditure per capita tripled from 1991 to 2008, reflecting an expansion of social protection programs for vulnerable Colombians [26]. Since 2000, several poverty reduction programs have been introduced which include conditional cash transfer programs, social housing, non-contributory pensions and food programs [27, 28]. We examine how the relationship between regional economic conditions and mortality changed in Colombia before and after 2000, when most of these programs were introduced.

## Methods

### Population counts

Data were obtained from the National Statistics Office [29]. Data on population counts for 5-year age groups, sex and region came from censuses and corresponding official demographic projections. Because data were only available since 1985, we performed additional demographic projections to obtain population counts for the years 1980–1984. Based on data from the national census of 1985 we ran a back-projection of the Colombian regional population by sex and 5-year age group [30] using the software PASEX (Population Analysis System) developed by the of the United States Census Bureau [31]. This program interpolates between two population age structures. The values of age-sex-specific population data for years not given as input are linearly interpolated between input values, and values before the first input value and values after the last input value are held constant at the level of the nearest input value [32]. Additional details on the procedure are available elsewhere [32].

### Mortality data

Data on deaths between 1980 and 2010 were collected and harmonized by the National Statistics Office for all regions based on international guidelines [29]. Information on sex, age and year of death were missing for 7.6 % of all deaths (289,429 out of 3,796,029 deaths). We excluded these deaths from the analysis because of lack of sufficient information to perform multiple imputations.

Data on death and population counts were grouped and linked by region, year, sex and 5-year age group combination from 1980 onwards [29]. We used death and population counts to obtain crude mortality rates for every 5-year age group, sex, year and region combination for the periods 1980–1995 and 2000–2010. Following the approach of earlier studies [10, 12, 18, 19], we then age-standardized mortality rates using the WHO standard population of 1998 in order to take into account changes over time in the population age structure [33].

### Regional GDP per capita

Data on regional GDP in constant Colombian Pesos (COP) of 2005 were obtained from the National Statistics Office [34]. We chose GDP as an indicator of regional economic conditions because it was the only regional economic indicator with complete and comparable data for a sufficiently extended period. Other indicators disaggregated by region such as the unemployment rate were only available for recent years. Regional GDP per capita (GDPpc) was obtained by dividing yearly regional GDP over the total regional population. Information on GDPpc was in principle available for three separate series: 1980–1995, 1990–2005 and 2000–2010. Although the correlation between the series in the overlapping years was very high, existing differences in the method used by National Statistics to estimate GDP prevented us from merging the three series. For this reason we performed the analysis separately for the first (1980–1995) and last series (2000–2010). The end of the first series coincides with the passing of the health care reform law in 1993 [24], while the second series coincides with the start of major decentralization reforms to transfer national resources to the regions, a rapid expansion of health insurance coverage [24] and the introduction of poverty reduction programs [27].

Our approach exploits variations over time in economic output within each region. From 1980 to 1990, Colombia was divided into 33 administrative regions: 23 departments, the District Capital of Bogota and nine independent territories (one archipelago and eight extensive and sparsely-populated territories of plains and forests). The National Statistics Office reports population and mortality statistics separately for each of these regions, except for the independent territories, which are grouped together albeit differently across the periods studied: From 1980 to 1995, statistics are reported for 25 regions (the district capital, 23 regions, and all the former independent territories grouped). From 2000 to 2010, statistics are reported separately for 29 regions (the district capital, 27 regions, and the Amazonia region, which encompasses five former independent territories). This implies we have a slightly different number of regions for each series. In sensitivity analyses, however, we found that excluding units that were differently grouped across period yielded virtually the same results as those presented here.

### Methods of analysis

We implemented ordinary least squares (OLS) regression models with the natural logarithm (log) of the annual mortality rate (per 100,000) as the dependent variable and the log of regional GDPpc as the key independent variable. Following the approach used in previous studies [1], we applied a region fixed effect model stratified by sex and age groups to examine how changes in regional GDPpc were associated with changes in mortality. The basic model specification is as follows:

1 $$ \boldsymbol{Log}{\left(\frac{\boldsymbol{Deaths}}{\boldsymbol{Population}}\right)}_{\boldsymbol{j}\boldsymbol{t}}=\kern0.5em {\propto}_{\boldsymbol{t}}+{\boldsymbol{\beta}}_1{\boldsymbol{X}}_{\boldsymbol{j}\boldsymbol{t}}+{\boldsymbol{\beta}}_2\boldsymbol{LogGDPp}{\boldsymbol{c}}_{\boldsymbol{j}\boldsymbol{t}}+{\boldsymbol{\beta}}_3\boldsymbol{Regio}{\boldsymbol{n}}_{\boldsymbol{j}}+{\boldsymbol{\beta}}_4\boldsymbol{Regio}{\boldsymbol{n}}_{\boldsymbol{j}}*\boldsymbol{Year}+{\boldsymbol{\varepsilon}}_{\boldsymbol{j}\boldsymbol{t}} $$

where *j* denotes region and *t* year, $Log\left(\frac{Deaths}{Population}\right)$ is the natural logarithm of the age-adjusted mortality rates; *X* is a vector of regional socio-demographic controls (college enrolment, health insurance coverage and transfers from central government to regions); *LogGDPpc* is the logarithm of regional GDPpc; *Region* is a fixed-effect for each region, ∝ is a vector of year fixed effects; *R* is a region-specific intercept; Region*Year is a region-specific linear time trend; and *ɛ* is the error term. The year effect controls for factors that vary uniformly across regions over time, while region fixed effects control for time-invariant factors that differ across regions. This model effectively controls for all time-invariant differences among regions. We clustered standard errors by region to obtain unbiased standard errors in the presence of serial correlation. Following the approach of previous studies, we weighted models by the square root of population to account for heteroskedasticity [1].

The association between regional economic conditions and mortality is identified out of variations in GDPpc over time within a given region relative to changes in other regions, controlling for national trends as well as region-specific linear time trends. The purpose of this strategy is to identify the impact of the business cycle, namely the repeated sequences of economic expansions and contractions, rather than the impact of economic growth. By incorporating region and year fixed effects as well as regional linear trends our model captures the cyclical component from the increasing secular trend in the log of GDP for each region. Estimates can therefore be interpreted as the impact of regional annual deviations from the linear regional trend in GDPpc on annual deviations in mortality.

Following the specifications of previous studies [1, 2, 6, 7, 11] we implemented models separately for three age groups: 20–44 (representing the young adult population), 45–64 (middle aged working individuals), and 65+ (corresponding to the senior population). To test whether there was a significant difference in the association between business cycles and mortality between the two periods, we pooled data into a single series and incorporated an interaction term between period and GDPpc, allowing for interactions between all control variables and period.

### Assessing the impact of mortality under-registration

A common concern with data on mortality in low- and middle-income countries is under-registration [35], which varies across Colombian regions and has generally improved over time [36]. In order to test the effect of under-registration on our estimates, we carried out analyses in a restricted sample of years for which levels of registration were 70 % or higher across all age and sex groups in each region. To identify levels of registration for each region, we followed the approach proposed by the Pan American Health Organization [37] and previously applied in Colombia [36, 38]. This approach estimates the expected number of deaths for each region and year based on inter-censual changes in population. In a first step, life tables including yearly number of deaths by 5-year age groups, sex and region were calculated for each region for the census years 1985, 1993, and 2005 [39]. Using the cohort component method, the mid-year populations were projected forward for the years 1987, 1992, 1997, 2002, and 2007. In a second step, based on the mortality rates obtained from the projected mid-year populations (and the most recent life-table), we used linear extrapolation to calculate the expected number of deaths for each year, 5-year age group, sex and region. Registration levels were calculated based on the ratio of registered deaths (according to the National Statistics Office) to expected deaths (based on the inter-censual changes in population) for each year, 5-year age group, sex and region.

### Additional control variables

To test the robustness of our results to factors other than the economy (which varied over time across regions), we incorporated the following time-varying confounders for each region: college enrolment (percentage of enrolled students among the population aged 16–24) [40], the percentage of population with government-subsidized health insurance [41], and the percentage of population with contributive health insurance [41]. Furthermore, we controlled for yearly financial transfers for health from the central government to each region entered in the model as the log of constant Colombian pesos (COP) in 2005 [42]. Health transfers include funds transferred from the national government for increasing subsidized health insurance coverage, public health programs and to address the needs of the uninsured population. These transfers, however, only represent a part of the total funding for health expenditures in each region, which also include funding from regional budgets. Unfortunately, there are no detailed data on regional funding allocations for health care. These variables were chosen because they are potentially associated with mortality and not directly related to the business cycle. Unfortunately, data on these variables were only available for the second period (2000–2010), which prevented us from incorporating these controls in analyses for the period 1980–1995. However, results for the years 2000–2010 indicate that controlling for these variables had only a very small impact on the estimates. Table 1 provides an overview of the exact definitions, source and years covered for the variables used in the models.

**Table 1:** Description of the yearly regional variables used in the models, Colombia 1980–2010

Variable	Period available	Units	Source
Registry of deaths [29]	1979–2012	Number of deaths	National Office of Statistics [DANE]
Population (censuses and estimations) [29]	1985–2020	Inhabitants	
GDP per capita [51]	1980–1995 and 2000–2013	Constant 2005 Colombian Pesos (COP)	
Enrolment to college [40]	2000–2012	Percentage of enrolled students to post-secondary education among the population aged 16–24	Ministry of education
Subsidized regime – affiliated population [41]	1995–2010	Percentage of population insured in the subsidized scheme over total population	National Department of Planning [DNP]
Contributive regime – affiliated population [41]	1996–2010	Percentage of population insured in the contributive scheme over total population	
Transfers to health [42]	1994–2013	Constant 2005 Colombian Pesos (COP)	

For comparability with earlier studies [1, 12], we summarize results from models excluding regional trends (Table 2). In our case, however, regional linear trends are essential to capture the impact of yearly deviations from the average trend in GDPpc within each region, more closely measuring the business cycle. In addition, regional linear trends enable us to control for some of the unobserved regional variables that changed linearly over time and were not controlled for in the models. Importantly, regional linear trends may also capture some of the effect of secular improvements in under-registration, minimizing bias that these changes may introduce in the relationship between changes in GDPpc and mortality. As in previous studies [1, 12], we base our interpretation on estimates with linear trends, as these are considered a more stringent specification relative to models without linear trends. All analyses were conducted in SAS® version 9.2.

**Table 2:** Association between regional Gross Domestic Product (GDP) per capita and all-cause mortality for age groups excluding regional linear trend, Colombia, 1980–2010

	Men			Women		
	Estimate	SE	*p* value	Estimate	SE	*p* value
20–44 years						
Men	−0.0031	0.0149	*0.83*	−0.0014	0.0034	*0.68*
Women	−0.0060	0.0088	*0.50*	0.0015	0.0020	*0.45*
45–64 years						
Men	0.0064	0.0149	*0.67*	0.0055	0.0169	*0.75*
Women	−0.0007	0.0121	*0.95*	0.0082	0.0109	*0.45*
65+ years						
Men	0.0434	0.0976	*0.66*	−0.0211	0.0842	*0.80*
Women	0.0428	0.0820	*0.60*	0.1127	0.0586	*0.05*
Region dummies	Yes			Yes		
Year dummies	Yes			Yes		
Regional linear trends	No			No		

OLS estimates and robust standard errors (SE)

## Results

Table 3 shows means and standard deviations of regional age-standardized mortality rates by sex, age-group and period. As expected, mortality rates were higher for men than for women, and they increased steadily with age. Mortality rates decreased from the first period to the second for each sex and age group. The Table also shows means and standard deviations of regional per capita GDP and control variables. Average regional per capita GDP increased by about 70 % between the first and the second period. Colleague enrollment rates were around 17 % in both periods, and on average around 75 % of the population in each region had some form of health insurance.

**Table 3:** Descriptive statistics for the periods 1980–1995 and 2000–2010, ages >20 years, Colombia

	1980–1995		2000–2010	
	Mean	Standard deviation	Mean	Standard deviation
Response variable:				
Mortality rates (per 100,000 population)*				
Men				
20–44 years	75.59	11.22	68.19	12.21
45–64 years	201.39	8.59	158.70	15.07
65+ years	1009.54	68.79	932.02	18.43
Women				
20–44 years	24.26	2.65	18.58	1.60
45–64 years	141.38	10.31	98.76	8.81
65+ years	824.00	54.68	743.92	13.97
Explanatory variables:				
Economic conditions				
GDP per capita – year (constant thousands of 2005 COP: Colombian Pesos)	4420	1634	7381	5022
Control variables				
College enrollment rate			17.9 %	17.2 %
Percentage of affiliation to subsidized scheme			47.7 %	19.2 %
Percentage of affiliation to contributive scheme			26.6 %	13.6 %
Health transfers (constant million of 2005 COP)			101235	0.0022

*Average age-standardized mortality rates for each sex and age group separately by period

**Fig. 1:**
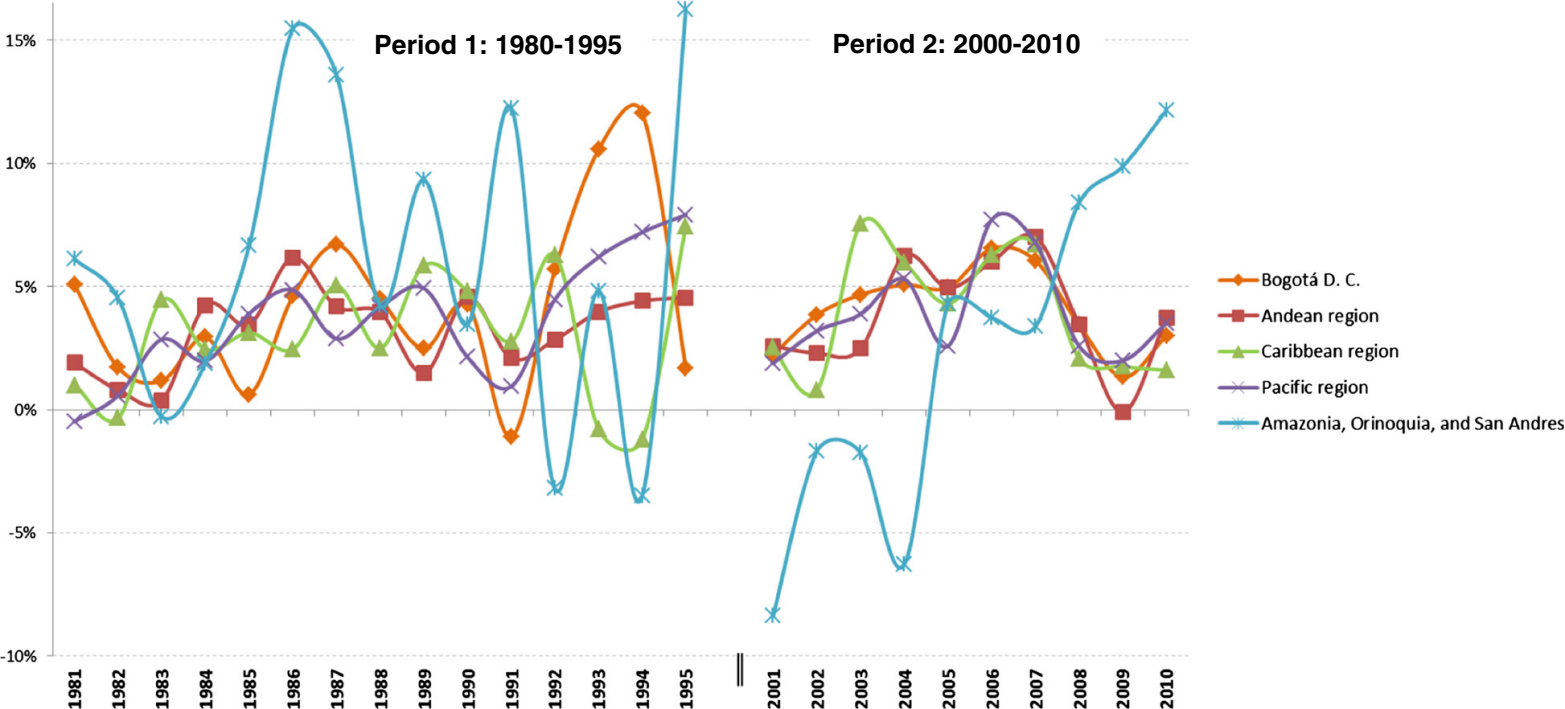
National trends of total GDP growth among major regions - Colombia (1981–1995, 2001–2010). The capital of the country, Bogota DC, is one region. The Andean region encompasses Antioquia, Boyacá, Caldas, Cundinamarca, Huila, Norte de Santander, Quindío, Risaralda, Santander, and Tolima. The Caribbean region encompasses Atlántico, Bolívar, Cesar, Córdoba, La Guajira, Magdalena, and Sucre. The Pacific region is Cauca, Chocó, Nariño, and Valle del Cauca. The group ‘Amazonia, Orinoquia, and San Andres’ encompasses Arauca, Casanare, Putumayo, San Andrés y Providencia (Archipelago), Meta, Caquetá, Amazonas, Guainía, Guaviare, Vaupés, and Vichada

Figure 1 shows growth in GDPpc during the years 1980–2010 for the five largest regions in Colombia. While there are common periods of recessions and booms, there were large variations in economic output across these regions, suggesting that there is sufficient variation to identify the effect of GDPpc on mortality.

Table 4 shows estimates from equation 1 for mortality at ages 20–44 separately by sex. As the results suggest, GDPpc was not significantly associated with mortality at ages 20–44 in the period 1980–1995 (column 1). In contrast, during the period 2000–2010, a one-point percentage increase in GDPpc was associated with a 0.03 % decline in male mortality at ages 20–44, and a 0.005 % decline in female mortality in the same age group (column 2). For women this effect was not significant at the 0.05 level when controlling for regional level confounders (column 3), while the effect was significant for men when including all controls. Table 5 shows results of identical models for mortality at ages 45–64. Although the sign of the coefficients is negative in tendency, estimates were not significant suggesting that regional GDPpc was unrelated to mortality in both periods.

**Table 4:** Association between regional Gross Domestic Product (GDP) per capita and all-cause mortality at ages 20–44, Colombia, 1980–2010

	1980–1995			2000–2010					
20–44 years	Model 1			Model 1			Model 2		
	Estimate	SE	*p* value	Estimate	SE	*p* value	Estimate	SE	*p* value
Men									
Log GDP per capita	−0.0053	0.0100	*0.59*	−0.0272	0.0119	*0.02*	−0.0246	0.0114	*0.03*
College enrollment rate							0.0152	0.0132	*0.25*
% subsidized insurance							−0.0038	0.0135	*0.78*
% contributive insurance							0.0216	0.0214	*0.31*
Health transfers (log)							0.0232	0.0134	*0.08*
Women									
Log GDP per capita	−0.0024	0.0022	*0.26*	−0.0049	0.0024	*0.05*	−0.0043	0.0024	*0.07*
College enrollment rate							−0.0001	0.0017	*0.93*
% subsidized insurance							0.0022	0.0024	*0.36*
% contributive insurance							0.0085	0.0049	*0.08*
Health transfers (log)							0.0039	0.0015	*0.01*
Region dummies	Yes			Yes			Yes		
Year dummies	Yes			Yes			Yes		
Regional linear trends	Yes			Yes			Yes		

OLS estimates and robust standard errors (SE)

**Table 5:** Association between regional Gross Domestic Product (GDP) per capita and all-cause mortality at ages 45–64, Colombia, 1980–2010

	1980–1995			2000–2010					
45–64 years	Model 1			Model 1			Model 2		
	Estimate	SE	*p* value	Estimate	SE	*p* value	Estimate	SE	*p* value
Men									
Log GDP per capita	−0.0227	0.0155	*0.14*	−0.0038	0.0137	*0.78*	−0.0049	0.0126	*0.70*
College enrollment rate							0.0173	0.0091	*0.06*
% subsidized insurance							−0.0011	0.0145	*0.94*
% contributive insurance							0.0234	0.0262	*0.37*
Health transfers (log)							−0.0010	0.0136	*0.94*
Women									
Log GDP per capita	−0.0106	0.0147	*0.47*	−0.0011	0.0085	*0.90*	−0.0016	0.0081	*0.84*
College enrollment rate							0.0063	0.0057	*0.27*
% subsidized insurance							0.0136	0.0080	*0.09*
% contributive insurance							−0.0028	0.0249	*0.91*
Health transfers (log)							−0.0005	0.0055	*0.93*
Region dummies	Yes			Yes			Yes		
Year dummies	Yes			Yes			Yes		
Regional linear trends	Yes			Yes			Yes		

OLS estimates and robust standard errors (SE)

Table 6 summarizes the results for mortality at ages 65 and older. From 1980 to 1995, a one-point percentage increase in GDPpc was associated with a 0.17 % reduction in old age male mortality (-0.1659, *p* = 0.04). A similar effect was observed for females, although estimates were not significant at the 0.05 level (-0.1115, *p* = 0.09). In contrast, in the more recent period (2000–2010), none of the estimates were significant, suggesting that there was no relationship between regional GDPpc and mortality from 2000 to 2010.

**Table 6:** Association between regional Gross Domestic Product (GDP) per capita and all-cause mortality at ages 65+, Colombia, 1980–2010

	1980–1995			2000–2010					
65+ years	Model 1			Model 1			Model 3		
	Estimate	SE	*p* value	Estimate	SE	*p* value	Estimate	SE	*p* value
Men									
Log GDP per capita	−0.1659	0.0820	*0.04*	0.0560	0.0454	*0.22*	0.0515	0.0474	*0.28*
College enrollment rate							−0.1363	0.0444	*0.00*
% subsidized insurance							0.0707	0.0585	*0.23*
% contributive insurance							0.1683	0.1373	*0.22*
Health transfers (log)							0.0054	0.0380	*0.89*
Women									
Log GDP per capita	−0.1115	0.0667	*0.09*	0.0557	0.0514	*0.28*	0.0427	0.0526	*0.42*
College enrollment rate							−0.0144	0.0317	*0.65*
% subsidized insurance							0.1365	0.0600	*0.02*
% contributive insurance							0.1901	0.1303	*0.14*
Health transfers (log)							−0.0059	0.0283	*0.84*
Region dummies	Yes			Yes			Yes		
Year dummies	Yes			Yes			Yes		
Regional linear trends	Yes			Yes			Yes		

OLS estimates and robust standard errors (SE)

To assess whether the association between GDPpc and mortality changed over the two periods, we pooled data for both series and implemented a set of models that included interaction terms between period and each of the variables in the models. Table 7 shows the estimates of the interaction between period and GDPpc. There was no interaction between GDPpc and period for mortality at younger (20–44) or middle-ages (45–64). In contrast, there was a significant and positive interaction between period and GDPpc for mortality at older ages among men (0.233, *p* = 0.02) and women (0.213, *p* = 0.01). This suggests that old-age mortality shifted over time from being countercyclical in 1980–1995 to being essentially unrelated to economic conditions in 2000–2010.

**Table 7:** Association between regional Gross Domestic Product per capita (GDPpc) and all-cause mortality between periods, interaction term, Colombia, 1980–2010

	Men			Women		
	Estimate	SE	*p* value	Estimate	SE	*p* value
20–44 years						
Log GDPpc * Period	−0.0124	0.0202	*0.54*	−0.0018	0.0044	*0.69*
Log GDPpc (Period 1)	−0.0044	0.0101	*0.66*	−0.0024	0.0022	*0.26*
45–64 years						
Log GDPpc * Period	0.0168	0.0254	*0.51*	0.0167	0.0189	*0.38*
Log GDPpc (Period 1)	−0.0230	0.0156	*0.14*	−0.0107	0.0145	*0.46*
65+ years						
Log GDPpc * Period	0.2327	0.1017	*0.02*	0.2126	0.0774	*0.01*
Log GDPpc (Period 1)	−0.1665	0.0820	*0.04*	−0.1125	0.0664	*0.09*
Region dummies	Yes			Yes		
Year dummies	Yes			Yes		
Regional linear trends	Yes			Yes		

OLS estimates and robust standard errors (SE); estimates are for the impact of a one-point increase in the log of GDPpc on mortality; variables included in each model are listed but their estimates are omitted from table

The variable Period was coded 0 for the years 1980–1995 and 1 for the years 2000–2010. The coefficient for the term ‘Interaction: Log GDPpc * Period’ thus refers to the interaction between the variables Log GDPpc and Period. The coefficient for the variable Log GDPpc refers to the effect of GDPpc on mortality in the first period (1980–1995)

### Robustness checks

A potential concern is that improvements in the coverage of death registration over time may be driving some of the relationships between GDPpc and mortality rates. While some of these secular improvements in death registration coverage are captured by regional linear trends and time fixed effects, if GDPpc was related to death coverage registration, this would result in biased estimates of the relationship between regional GDPpc and mortality. To assess the impact of this potential bias, we conducted a robustness checks with a restricted sample of years in each region for which registration levels were 70 % or higher across all age and sex combinations.

**Fig. 2:**
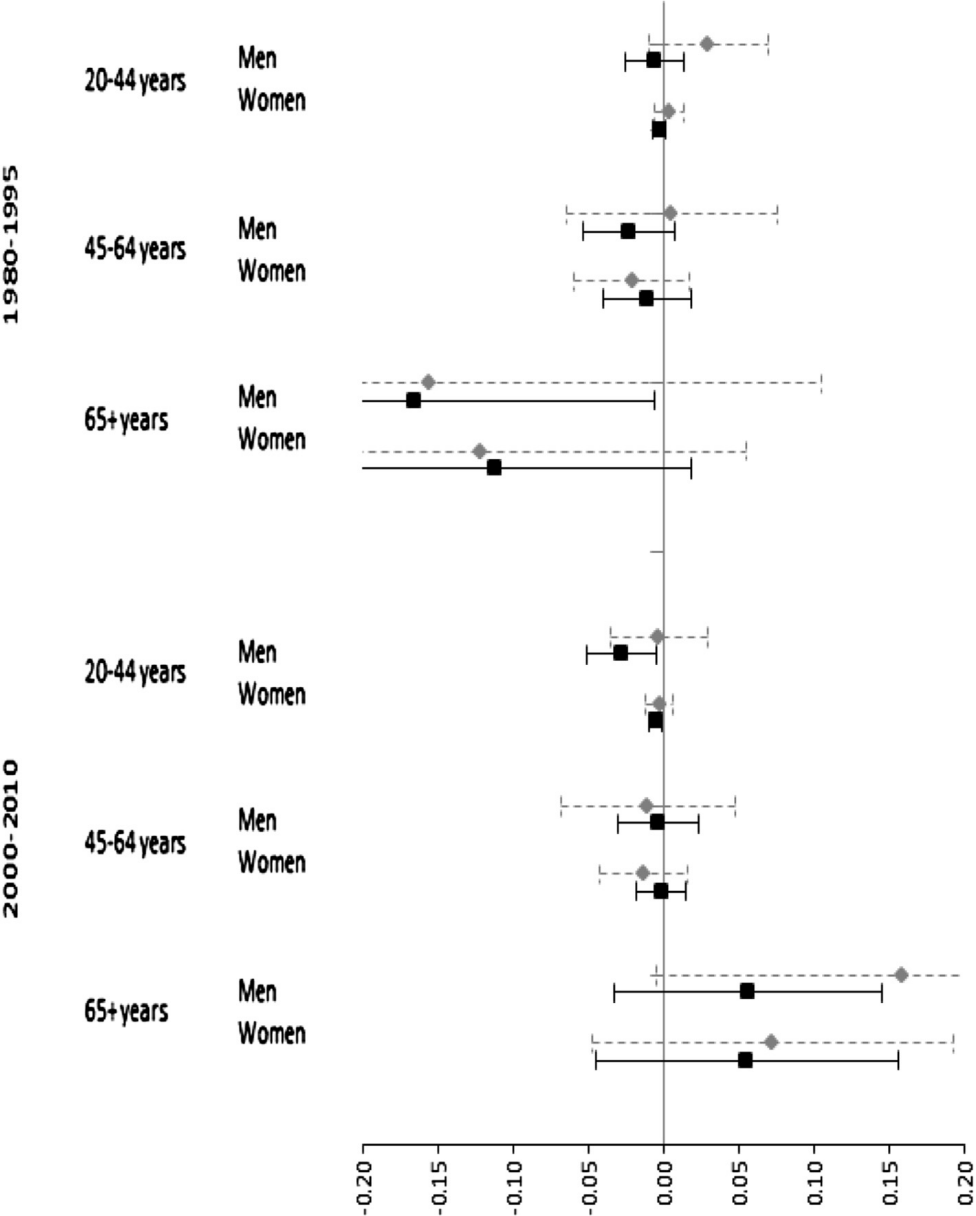
Association between regional Gross Domestic Product (GDP) per capita and all-cause mortality by sex and age groups, including 95 % confidence intervals, Colombia, 1980–2010. Note: Black squares and continuous lines are the estimates and CIs for the whole country (25 regions in 1980–1995 and 29 regions in 2000–2010). Grey diamonds and dotted lines represent the estimates and CIs of the models with those regions with registration levels above 70 % (12 and 13 regions, respectively). [*Icon for black squares and continuous lines*]: "Full sample of all regions" and [*Icon for grey diamonds and dotted lines*]: "Restricted sample (regions with registration above 70 %)"

Table 8 shows overall average levels of registration for each region across all years, sex and age groups in each period. Regions with no years with levels of registration of at least 70 % in all age and sex groups are excluded from the restricted sample. The final sample includes 12 regions in period 1 and 13 regions in period 2. Table 8 shows that coverage of death registration gradually increased over the study period. Between 1980–1995 and 2000–2010, average levels of registered deaths increased from 60.8 to 73.1 %. At the same time, levels of registration improved in 18 out of 25 regions over time. As a sensitivity analysis, Table 9 shows the results of models that restrict the sample to years of coverage in the registration of deaths of at least 70 % in all sex and age groups in a given period. For comparison purposes, estimates from this restricted sample are presented alongside estimates for the full sample of all regions and years. To better illustrate differences, Fig. 2 also plots estimates from Table 9 and incorporates 95 % Confidence Intervals for each estimate.

**Table 8:** Average levels of registration of the mortality database for all regions, Colombia, 1980–2010

Region	1980–1995	2000–2010
Antioquia	43.6 %	47.7 %
Atlántico	70.4 %	83.7 %
Bogotá	89.4 %	97.7 %
Bolívar	69.7 %	69.6 %
Boyacá	93.8 %	86.2 %
Caldas	87.0 %	95.4 %
Caquetá	59.1 %	72.5 %
Cauca	72.2 %	74.3 %
Cesar	48.7 %	80.0 %
Córdoba	48.4 %	64.5 %
Cundinamarca	79.3 %	86.3 %
Chocó	44.0 %	58.7 %
Huila	90.2 %	91.5 %
La Guajira	25.9 %	47.6 %
Magdalena	48.6 %	79.7 %
Meta	54.9 %	84.9 %
Nariño	79.1 %	83.0 %
Norte de Santander	85.5 %	86.6 %
Quindío	76.4 %	89.2 %
Risaralda	87.4 %	95.6 %
Santander	86.6 %	90.1 %
Sucre	56.5 %	68.4 %
Tolima	72.0 %	83.3 %
Valle	89.9 %	98.2 %
Arauca		83.6 %
Casanare		59.4 %
Putumayo		60.8 %
San Andrés y Providencia Archipelago		48.8 %
Amazonía		55.6 %
Independent territories	36.0 %	
Colombia	60.8 %	73.1 %

(i) Overall, coverage of death registration gradually increased over the study period. Between 1980–1995 and 2000–2010, average levels of registered deaths increased from 60.8 to 73.1 %. At the same time, levels of registration improved in 18 out of 25 regions over time. The restricted subsample includes 12 regions in period 1 (Bogotá, Boyacá, Caldas, Caquetá, Cauca, Huila, Nariño, Norte de Santander, Quindío, Risaralda, Santander, and Valle) and 13 regions in period 2 (Atlántico, Bogotá, Boyacá, Caldas, Cundinamarca, Huila, Meta, Norte de Santander, Quindío, Risaralda, Santander, Valle, and Arauca). (iii) We allowed regions with levels of registration higher than 70 % in some -but not all- of the years to contribute to the restricted sample, but only for the years in which they had registration of 70 % of higher. For example, in period 1 Bogota had registration coverage above 70 % for years 1980–1994, but not in 1995. We therefore included only years 1980–1994 for Bogota and excluded 1995. (iv) Even if some regions had average levels of registration higher than 70 %, they had no years for which registration levels were above 70 % in all sex and age groups for at least one year, and therefore were not included in the restricted sample, e.g., Atlántico in the first period.(v) Likewise, some regions had average levels of registration lower than 70 % (e.g., Caquetá, 44 % in the first period), yet they had at least one year for which registration was higher than 70 % in all sex and age groups, and were therefore part of the restricted sample in those years

**Table 9:** Sensitivity analysis for under-registration for the association between regional Gross Domestic Product (GDP) per capita and all-cause mortality for sex and age groups, Colombia, 1980–2010

Log GDP per capita	1980–1995						2000–2010					
	Full			Registration > =70 %			Full			Registration > =70 %		
	25 regions			12 regions			29 regions			13 regions		
	Estimate	SE	*p* value	Estimate	SE	*p* value	Estimate	SE	*p* value	Estimate	SE	*p* value
20–44 years												
Men	−0.0053	0.0100	*0.59*	0.0299	0.0203	*0.14*	−0.0272	0.0119	*0.02*	−0.0028	0.0167	*0.87*
Women	−0.0024	0.0022	*0.26*	0.0038	0.0050	*0.44*	−0.0049	0.0024	*0.05*	−0.0025	0.0048	*0.61*
45–64 years												
Men	−0.0227	0.0155	*0.14*	0.0055	0.0358	*0.88*	−0.0038	0.0137	*0.78*	−0.0100	0.0293	*0.73*
Women	−0.0106	0.0147	*0.47*	−0.0208	0.0196	*0.29*	−0.0011	0.0085	*0.90*	−0.0128	0.0150	*0.39*
65+ years												
Men	−0.1659	0.0820	*0.04*	−0.1554	0.1328	*0.24*	0.0560	0.0454	*0.22*	0.1585	0.0830	*0.06*
Women	−0.1115	0.0667	*0.09*	−0.1216	0.0905	*0.18*	0.0557	0.0514	*0.28*	0.0728	0.0614	*0.24*

OLS Estimates and robust standard errors (SE). All models adjusted by region dummies, year dummies, and regional linear trends

The first point to note is that estimates from the restricted sample are less precise than estimates from the full sample, reflecting the smaller sample size in the restricted sample. A second point to note is that there are no significant differences between estimates for the restricted sample and estimates for the full sample for any of the sub-groups. In most cases, estimates for the restricted and full sample are in fact very close if one considers the uncertainty around some of these estimates. Nevertheless, there are two exceptions in the second period: the estimate for men ages 20–44 is negative and significant for the full sample, but it is close to zero and non-significant in the restricted sample. For males ages 65+ (bottom bars of the Figure, left Panel), the estimate is positive but not significant in the full sample; while it is also positive in the restricted sample, the estimate is larger and approaches statistical significance at the 0.05 level for the restricted sample. For all other sub-groups, estimates for the restricted and full sample are in fact very close.

## Discussion

### Summary

The purpose of this study was to assess the relationship between business cycles and mortality in Colombia during two periods. Contrary to some studies in high-income countries, we found no evidence of procyclical mortality in Colombia. We found some evidence that mortality at older ages was countercyclical from 1980 to 1995, but there was no relationship between GDPpc and mortality for older ages from 2000 to 2010, suggesting that old age mortality may have changed from being countercyclical to being unrelated to the business cycle. Likewise, mortality at ages 20–44 was unrelated to GDPpc from 1980 to 1995, but it was countercyclical from 2000 to 2010, although the two estimates were not significantly different from each other.

### Explanation of results

Our findings for mortality at the younger ages (20–44), and especially for men, contrast with results from most studies in high-income countries suggesting that mortality for this age group is generally procyclical [1, 2, 5, 8]. This discrepancy may be due to differences in the distribution of causes of death. However, in the US, for example, pro-cyclical mortality is especially pronounced for mortality from cardiovascular disease, homicide and (vehicle) accidents, which are also leading causes of death in Colombia [43]. Another explanation for the discrepant findings is a difference in the association between business cycles and specific causes of death. Our analysis only focused on total mortality given the poor quality of data on specific causes, but a potential hypothesis is that mortality from these leading causes of death is not procyclical in Colombia. Differences between results for Colombia and high-income countries may also be due to differences in the period of study, as well as the fact that we use GDP per capital as indicator of economic conditions, while several studies in the US and Europe use unemployment rates.

We found that the economic expansions were associated with decreased old age mortality from 1980 to 1995, whereas old-age mortality was unrelated to the regional economy from 2000 to 2010. This finding is in line with recent evidence that the association between the business cycle and mortality shows some instability over time. For the US, recent findings by Ruhm [12] suggest that a potential explanation for the emergence of counter-cyclical cancer mortality in recent years is the increasing importance of financial resources in receiving sophisticated and expensive therapies. In Colombia, financial resources may have been more important to access sophisticated and expensive therapies in the first period, during which health insurance coverage was limited. In contrast, in the second period, the expansion of health insurance coverage [24, 25] implies that individuals may more easily have access to these therapies irrespective of the business cycle. This may explain the shift from countercyclical to acyclical mortality for older males between the first and second period.

Our findings are at odds with a previous study showing that infant mortality in Colombia increased when economic conditions improved [44]. However, our study focused on mortality at ages 20 years and above, which may show a different association with business cycles than infant mortality. The finding that infant mortality is pro-cyclical has been shown to be partly attributable to selection (compositional changes in the pool of mothers conceiving during recessions and booms). For example, in the US, African-American mothers of children born during times of high unemployment tend to be more educated than African-American mothers of children born during low unemployment, which contributes to lower mortality during recessions [45]. While changes in behavior may also be part of the mechanism leading to lower infant mortality during recessions, this illustrates the fact that the mechanisms underlying the relationship between business cycles and mortality may differ by age and cause of death.

Although for most sub-groups our analyses restricted to regions with higher levels of registration yielded similar estimates as those for the full sample, differences in estimates for young (20–44) and older men (65+) deserve some explanation. Some of this difference may be due to the smaller sample size and higher standard errors in the restricted sample analysis. Nevertheless, it appears that the estimates for the restricted sample are in tendency more positive than estimates for the entire sample. This pattern may be due to compositional differences between the full and restricted sample. Overall, the restricted sample contains regions from all major geographical zones (Caribbean, Pacific, Andean, and Western plains), but regions in this restricted sample are slightly more affluent than regions excluded due to their lower registration levels. Average GPDpc is 15 to 22 % higher in regions with registration higher than 70 % as compared to the other regions (4841 thousand Pesos in the regions with higher registration versus 4180 thousand Pesos in those with lower registration-levels in the first period, versus a difference of 8262 thousand Pesos to 6759 thousand Pesos in the second period). This may imply that estimates from the restricted sample capture the relationship between business cycles and mortality in regions that are at a relatively higher level of economic development. This is consistent with previous evidence suggesting that mortality is procyclical in highly developed regions but countercyclical in less developed regions within Mexico [15].

### Limitations

Some limitations should be considered in our study. A major concern is the under-registration of mortality in many regions of Colombia [36], which we addressed by restricting the sample in sensitivity analyses to regions that had relatively high registration coverage in all years (Table 9). These results yielded mixed results. On the one hand, although standard errors are very large, estimates were in tendency similar to those we observed for the full sample in two ways: first, we found no evidence of procyclical mortality in any group or period as it has been observed for high-income countries. Second, there is an indication of a changing relationship between GDP and mortality at ages 65+ between the first and the second period. On the other hand, the large uncertainly around these estimates suggest that some caution should be exercised given the potential that changes in under-registration might remain important. Although we found no correlation between the business cycles and rates of under-registration, estimates of under-registration may be imperfect and a full assessment requires a more detailed study.

We used GDPpc as a proxy for macro-economic conditions in our study. Unfortunately, there are no reliable time series on unemployment rates at the regional level covering sufficiently extended periods. Estimates are therefore not directly comparable to estimates from earlier studies in high-income countries which primarily have used unemployment rates as indicators of macro-economic conditions [2, 8, 10, 11, 18], sometimes controlling for GDP [1, 4, 5]. Yet, while unemployment rates may be the preferred measure of the business cycle in high-income countries, unemployment rates are often considered a poor measure of the business cycle in less developed countries. Similar to their Mexican counterparts [15], Colombian workers can experience changes in earnings but continue to be classified as employed because of the large share of the work force in self-employment, and the fact that many laid-off workers quickly turn to self- or part-time employment in the absence of unemployment benefits [46]. This relates also to the fact that a large share of workers are in the informal sector (60 % in 2009 [47]) [46, 48], with a changing proportion over time, making it difficult to use a common definition of unemployment over an extended period. Although informal sector [49]. Informal sector workers lack regular social benefits and do not contribute social security contributions [46], they represent an important share of the Colombian economy making it difficult to quantify in unemployment statistics based on survey data.

We incorporated controls for regional variables such as college enrolment, health insurance coverage and transfers from central government to regional areas. Unfortunately we were only able to obtain reliable data on these regional variables for the period 2000–2010 (see Table 1). As a result, we were unable to directly control for regional factors that may have affected mortality in the period 1980–1995. However, we expect fixed effects for calendar years to control for unmeasured confounders that varied systematically across all regions. In addition, region-specific time trends control for factors linearly associated with mortality in each region. Although we cannot discard the possibility that unmeasured factors could have influenced our results, it is reassuring that associations for the period 2000–2010 were largely unchanged after incorporating a wider set of regional control variables.

We found that increasing coverage for subsidized health insurance as well as health transfers were associated with increased mortality at ages 45–64 (Table 5) and 65+ (Table 6). In the context of our region and year fixed effect models, this implies that regions that had a faster increase in subsidized health insurance coverage between 2000 to 2010 experienced higher mortality increases than regions that had slower increases in insurance coverage. Although this seems counterintuitive, the rates of expansion of subsidized health insurance as well as transfers from the government were selective with worse-off regions being the focus of larger efforts towards expanding coverage [49]. Increases in coverage may thus have been larger in less healthy regions, so that they do not necessarily reflect the causal impact of increasing insurance coverage. Thus, while useful as a control variable, it is difficult to interpret estimates of health insurance coverage in our models as evidence of a causal effect of increasing health insurance coverage. In fact, the existing evidence suggests that increased access to subsidized health insurance in Colombia is associated with reduced infant mortality [50] and improved adult health [17].

## Conclusions

Notwithstanding the limitations of registry data in low- and middle-income countries, our results suggest that contrary to some studies in high-income countries, there is no evidence of procyclical mortality in Colombia. In contrast, we find evidence that mortality at older ages was countercyclical from 1980–1995. However, business cycles and mortality appear to be unrelated in the more recent period also among the more affluent regions with better mortality registries.

## Abbreviations

GDP: Gross domestic product
GDPpc: Gross domestic product per capita
OLS: Ordinary least squares
